# Diabetes alters the supragingival microbiome through plasma-to-saliva migration of glucose and fructose

**DOI:** 10.1186/s40168-025-02256-x

**Published:** 2025-12-04

**Authors:** Akito Sakanaka, Masahiro Furuno, Asuka Ishikawa, Naoto Katakami, Moe Inoue, Shota Mayumi, Daiki Kurita, Hitoshi Nishizawa, Kazuo Omori, Naohiro Taya, Emiko Tanaka Isomura, Mashu Kudoh, Hiroki Takeuchi, Atsuo Amano, Iichiro Shimomura, Eiichiro Fukusaki, Masae Kuboniwa

**Affiliations:** 1 Department of Preventive Dentistry, Graduate School of Dentistry, The University of Osaka, Osaka, Japan; 2 Department of Biotechnology, Graduate School of Engineering, The University of Osaka, Osaka, Japan; 3 Department of Metabolic Medicine, Graduate School of Medicine, The University of Osaka, Osaka, Japan; 4 Japan Society for the Promotion of Science, Tokyo, Japan; 5 Department of Metabolism and Atherosclerosis, Graduate School of Medicine, The University of Osaka, Osaka, Japan; 6 Department of Diabetes Care Medicine, Graduate School of Medicine, The University of Osaka, Osaka, Japan; 7 Department of Oral and Maxillofacial Surgery, Graduate School of Dentistry, The University of Osaka, Osaka, Japan

**Keywords:** Type 2 diabetes, Dental caries, Saccharide migration, Oral microbiome, Supragingival biofilm, Salivary metabolomics, Shotgun metagenomics, Glycemic control

## Abstract

**Background:**

Dental caries, a dysbiotic biofilm disease driven by polymicrobial acidogenesis, often coexists with type 2 diabetes (T2D). Previous studies suggest covarying relationships between circulating and salivary metabolites in patients with T2D. However, the role of hyperglycemia-induced saccharide migration from plasma to saliva in caries pathogenesis remains unclear. Here, we developed a novel method for untargeted metabolomics profiling of trace saliva from sublingual and submandibular glands, comparing this profile with those of plasma and whole saliva in participants with T2D (*n* = 31) and those with normoglycemia (*n* = 30). This comparison aimed to determine how circulating saccharide migration into the oral cavity and its subsequent microbial consumption are linked to dental caries. Additionally, shotgun metagenomic sequencing was combined with this analysis to investigate the cariogenic impact of circulating saccharide migration on the composition and function of supragingival biofilm using MetaPhlAn4 and HUMAnN3 pipelines.

**Results:**

The metabolomics profiles of glandular saliva showed intermediate dissimilarity between plasma and whole saliva, reflecting cardiometabolic traits more sensitively than whole saliva. Glucose and fructose showed a decreasing positive correlation with glycemic parameters in the order of plasma, glandular saliva, and whole saliva, suggesting systemic-to-oral migration and subsequent microbial consumption. Saccharide migration was more pronounced in participants with dental caries and plaque accumulation, coinciding with shifts in supragingival microbiota, including depletion of *Streptococcus sanguinis*, *Corynebacterium durum*, and *Rothia aeria*, and enrichment of *Streptococcus mutans*, *Veillonella parvula*, and *Actinomyces* sp. *oral taxon 448*. Glycolytic potential increased at the community level. Improved glycemic control reduced fructose migration and mitigated dysbiosis, decreasing fructose phosphotransferase abundance and shifting the *S. mutans*–*S. sanguinis* balance. Experimental validation demonstrated that fructose promotes *S. mutans* dominance over *S. sanguinis* in dual-species biofilms.

**Conclusions:**

This study establishes saccharide migration as a metabolic driver of supragingival dysbiosis in T2D. The findings highlight the role of both glucose and fructose in caries pathogenesis and suggest that glycemic control could serve as an effective strategy as part of caries control.

Video Abstract

**Supplementary Information:**

The online version contains supplementary material available at 10.1186/s40168-025-02256-x.

## Background

Type 2 diabetes (T2D) encompasses complex pathological conditions characterized by persistent hyperglycemia, often resulting in cardiovascular diseases, kidney failure, and retinopathy. T2D arises from insulin resistance and impaired insulin secretion, with sedentary lifestyles and dietary changes as major risk factors. Currently affecting approximately 537 million adults worldwide, T2D is projected to reach 783 million by 2045, imposing significant public health and societal burdens [1].

T2D causes oral complications, most commonly dental caries and periodontal diseases with 2.3 billion and 796 million adults worldwide having untreated caries and severe periodontitis, respectively, in 2017 [2]. Despite a well-established causal relationship between T2D and periodontal diseases [3, 4], the effect of T2D on dental caries remains unclear. Dental caries is a dysbiotic biofilm disease driven by polymicrobial metabolic activity that promotes acidogenesis and mineralized tooth destruction [5, 6]. Frequent exposure to fermentable carbohydrates promotes spatially structured biofilms with extracellular glucan matrices and localized acidic microenvironments, favoring an acidogenic and aciduric microbial community [7, 8]. *Streptococcus mutans*, a notable example, exhibits acidogenic and aciduric traits and produces extracellular polysaccharides, contributing to the pathogenesis of caries [6, 9, 10]. Recent molecular analyses, however, have identified a new disease-associated community (e.g., *Actinomyces* spp., *Scardovia wiggsiae*, and *Selenomonas sputigena*) [11–13] and a health-associated community (e.g., *Streptococcus oralis*, *Streptococcus sanguinis*, and *Corynebacterium durum*) [14, 15]. Despite some debate, most epidemiological studies demonstrate an association between T2D and caries [16–19], with poorer glycemic control associated with a higher prevalence of caries in individuals with T2D [20, 21]. However, our understanding of the biological pathways underlying this association remains incomplete. The typical explanation involves reduced salivary flow and qualitative changes in saliva, impairing acid-buffering and remineralization capabilities [22], though mechanistic validation is often limited.

Recent research has shifted focus to diabetes-associated changes in salivary metabolites. Since salivary glands receive circulating components, saliva exhibits increased glucose levels in response to hyperglycemia [23–25]. Metabolomic profiling in patients with T2D also reveals covarying increases in circulating and salivary metabolites including multiple saccharides [26–29], which may fuel cariogenic bacteria, altering the composition and metabolic activity of the supragingival microbiome. However, existing evidence primarily derives from whole-saliva analyses, where microbial metabolism confounds the interpretation of host-derived metabolite dynamics. This obscures the true relationship between plasma metabolites and caries pathogenesis.

To address this, we have developed a novel method for untargeted metabolomic profiling of sublingual and submandibular gland-derived trace saliva, preserving intact metabolite profiles before oral microbial modification. The sublingual and submandibular glands produce the majority of unstimulated saliva [30], and their continuous secretion may influence the composition of the oral microbiota and affect the risk of oral disease. By comparing these profiles with plasma and whole-saliva samples from participants, including those with T2D, we demonstrate that hyperglycemia-induced saccharide migration into saliva fuels cariogenic bacteria. Using shotgun metagenomic sequencing, we further investigated the functional impact of saccharide migration on supragingival biofilms through MetaPhlAn4 and HUMAnN3 pipelines [31, 32]. This analysis reveals that hyperglycemia promotes glucose and fructose migration into the oral cavity, enriching cariogenic species and shifting biofilm metabolism toward glycolysis and carbohydrate degradation. Moreover, improved glycemic control reduces plasma-to-saliva saccharide influx—particularly fructose—while reversing dysbiosis of the supragingival microbiome and decreasing community-level fructose phosphotransferase abundance. These findings help unravel the role of plasma-to-saliva saccharide migration in caries pathogenesis and support glycemic control as an effective strategy to mitigate caries risk in diabetes.

## Methods

### Study participants

This study, approved by the human ethics committees of Osaka University Hospital (no. 16374–6) and Osaka University Dental Hospital (no. H28-E40-2), was conducted following the Declaration of Helsinki. Written informed consent was obtained from all participants. In the absence of prior effect-size estimates for glandular-saliva saccharide migration, a feasibility-based target of approximately 30 participants per group was adopted. Systemically healthy volunteers were identified from Osaka University members or patients visiting Osaka University Dental Hospital. After obtaining written informed consent, 30 consecutive individuals meeting eligibility criteria were enrolled as controls. Exclusion criteria included abnormal salivary function, such as history of xerostomia, salivary-gland disease such as Sjögren’s disease, or prior head-and-neck irradiation; use of antibiotics within the past 3 months; use of any prescription medication within the past 2 weeks; and diagnosis of systemic disease. Participants with T2D were patients hospitalized to receive a standardized 2-week intensive glycemic control program at Osaka University Hospital between 2017 and 2019, which was not limited to severely uncontrolled cases. T2D was diagnosed based on the criteria of the World Health Organization National Diabetic Group 2006 and/or currently receiving treatment for diabetes. Exclusion criteria included (1) inability to achieve strict glycemic control due to severe hypoglycemia or progressive diabetic retinopathy; (2) severe nephropathy (serum creatinine > 2.0 mg/dL); (3) acute infection, severe trauma, or active cancer; (4) pre- or postoperative examination periods; and (5) unsuitability as determined by the attending physician. During hospitalization, patients underwent comprehensive diabetes management following Japanese treatment guidelines [33]. This included intensive glycemic control, as well as management of blood pressure, dyslipidemia, and body weight. Physicians set glycemic targets based on the guidelines, typically aiming for fasting blood glucose levels below 7.2 mmol/L (130 mg/dL) and 2 h postprandial blood glucose levels below 10.0 mmol/L (180 mg/dL). This study involved no dental interventions or oral hygienic instructions. Clinical data and samples from patients with T2D were collected on days 2 (baseline) and 14 (post-treatment) of hospitalization. As day 1 involved admission without the initiation of glycemic therapy, day 2 was considered the baseline for pre-intervention glycemic control.

### Clinical data collection

Blood samples collected in the morning following an overnight fast were biochemically tested based on standard protocols. Samples designated for metabolomics analysis were immediately cooled at 4 °C and centrifuged, with the plasma stored at − 80 °C until analysis. Vital signs and body weight measurements were recorded concurrently.

Oral examinations were performed in a designated operatory equipped with two dental units at Osaka University Hospital by four calibrated dentists as previously described [28, 34]. Briefly, dental caries was assessed and defined using International Caries Detection and Classification System (ICDAS) criteria, with a score of 3 or higher indicating macroscopic tooth structure loss. The primary caries outcome was the per-participant count of teeth with an ICDAS score ≥ 3. For teeth with multiple lesions, the highest score was recorded, and each tooth was counted once. Root caries were recorded separately but excluded from the analyses, which focused on coronal lesions. All examiners completed the International Caries Classification and Management System (ICCMS™) e-learning and calibration training. Periodontal parameters, including probing depth, bleeding on probing, gingival recession, and clinical attachment level, were recorded at six sites per tooth and used to calculate the periodontal inflamed surface area (PISA) [35]. Supragingival biofilm accumulation was assessed at four sites per tooth using the Silness and Löe criteria [36]. The average score per participant was calculated as plaque index [37]. Tongue coating was scored as previously described [38].

### Glandular saliva sample collection

Prior to sample collection, filter papers were cut into 7 mm × 18 mm pieces using ceramic scissors and washed as follows: Each filter paper was placed in a pre-weighed 2 mL microcentrifuge tube (Eppendorf, Hamburg, Germany) and incubated in 1.5 mL of distilled water in a thermomixer (25 °C, 1000 rpm, 10 min). After removing the water, 1.5 mL of 20% ethanol was added to the tubes, which were then stored at − 30 °C. The day before sample collection, the filter papers were thawed at room temperature, the ethanol was discarded, and 1.5 mL of 99.5% ethanol was added. The papers were shaken (25 °C, 1000 rpm, 10 min), followed by ethanol evaporation at 65 °C. The tubes containing the filter papers were weighed to determine the filter paper weight.

Four calibrated dentists collected glandular saliva samples from the sublingual caruncles and folds, where the submandibular and sublingual glands drain. Before collection, a dental cotton roll was placed sublingually to remove residual saliva. A filter paper was placed sublingually using tweezers while participants maintained tongue-to-palate contact to avoid unintended areas (Supplementary Fig. 1). Once saturated, the filter paper was placed into a 2 mL glass vial (Nichiden-Rika Glass, Kobe, Japan) containing 530 µL of distilled water with an internal standard (ribitol, 0.2 mg/mL). This procedure was performed bilaterally, yielding two filter papers per vial. The vial’s weight was recorded pre- and post-collection to determine saliva volume.

The vial was shaken (25 °C, 1000 rpm, 10 min), subjected to ultrasonic treatment (40 kHz, 5 min), and centrifuged (4 °C, 4000 rpm, 3 min). After adding 1.4 mL of acetonitrile, the process was repeated, and 1.6 mL of the supernatant was transferred to a 2 mL tube. The supernatant was then dried with a vacuum concentrator (VC-96R; TAITEC, Koshigaya, Japan) (2000 rpm, 40 °C, 30 min), frozen in liquid nitrogen, and freeze-dried. The samples were stored at − 80 °C until analysis. Filter paper blanks underwent identical extraction. Weight measurements were taken to five decimal places using an analytical balance (GR-202; A&D, Tokyo, Japan). The air conditioning was turned off during measurements, and readings were taken once the values stabilized. Powder-free nitrile gloves were worn to prevent noise peaks during filter paper handling.

### Other sample collection

Four calibrated dentists collected additional oral samples. Participants were instructed to refrain from oral hygiene practices, including brushing, flossing, and mouthwash, and from eating or drinking (except water) for at least 1 h before sample collection. Unstimulated whole saliva was expectorated over 10 min into a 50 mL tube (Corning, Corning, NY, USA) kept on ice. After 15 min, the aqueous layer was collected, and samples with volumes of at least 1 and 0.1 mL were aliquoted into 2 mL tubes as study and quality control (QC) samples, respectively. These were then frozen in liquid nitrogen and stored at − 80 ℃ until analysis. Supragingival plaque samples were collected from the smooth surfaces of the teeth throughout the mouth using a Gracey curette (11/12 Mini Five; Hu-Friedy, Chicago, IL, USA). All plaque samples were frozen with liquid nitrogen and stored at − 80 °C until analysis.

### Metabolomic profiling

Glandular saliva samples were thawed at 4 ℃, dried using a lyophilizer for 30 min, and derivatized with a 20 mg/mL of methoxyamine hydrochloride solution in pyridine, followed by silylation with N-methyl-N-(trimethylsilyl)-trifluoroacetamide. The analysis was performed using a GC–MS/MS platform on the Shimazu GCMS-TQ8040 triple quadrupole mass spectrometer (Shimadzu, Kyoto, Japan) equipped with an AOC-20i autosampler (Shimadzu), a focus liner (GL Sciences, Tokyo, Japan), and an InertCap 5MS/NP ProGuard 2 M capillary column with a retention gap (0.25 mm × 32 m, 0.25 µm; GL Sciences), operated in a full MS scan mode. Each sample (1 µL each) was injected in a splitless mode. The helium flow rate through the column was set at 1.5 mL/min, with the column temperature set to 80 ℃ for 2 min, raised to 325 ℃ at 15 ℃/min rate, and held for 15 min. The transfer interface and ion source temperatures were set to 310 °C and 260 °C, respectively. Mass spectrometry was performed using electron impact ionization, with a selected mass range of 85–500 m/z. All samples analyzed were prepared in random order, and data were acquired in three batches. QC samples, comprising a homogeneous mix of all saliva samples, were injected every six biological samples for locally weighted scatter plot smoothing (LOWESS) normalization. A standard alkane mixture (C9-C40; GL Sciences) was also injected at the same interval to determine retention indices for peak identification. Decafluorotriphenylphosphine (Merck KGaA, Darmstadt, Germany) was added to the alkane mixture to monitor MS signal drift. Whole saliva and plasma samples were prepared and analyzed as previously described [28, 39].

### Metabolomics data processing

GC–MS data were converted into ABF format and processed using MS-DIAL (v.4.24) for feature detection, spectra deconvolution, metabolite identification, and peak alignment [40]. Normalization was then performed based on the internal standard (ribitol) and LOWESS algorithm. Signal drift over time was independently corrected by fitting a LOWESS curve to the MS signals measured in QC samples. The peak list was further normalized based on sample weight (g/mL). Metabolic features from blanks and those with a QC coefficient of variation exceeding 30% were discarded. After performing quality control, we detected 78, 143, and 125 annotated plasma, whole saliva, and glandular saliva hydrophilic metabolites using our GC–MS/MS platform, respectively.

### Metagenomic profiling

Plaque DNA was extracted using the Qiagen DNeasy PowerSoil Pro Kit (Qiagen, Hilden, Germany). Quality control was performed using a Qubit 2.0 fluorometer (Thermo Fisher Scientific, Waltham, MA, USA) and agarose gel electrophoresis with Agilent 2100 (Agilent Technologies, Santa Clara, CA, USA). Then, Illumina sequencing libraries were prepared using the NEBNext Ultra II DNA Library Preparation Kit for Illumina (NEB, Ipswich, MA, USA). Sequencing was conducted on the Illumina NovaSeq 6000 platform, targeting ~ 6 Gb of sequence per sample with 150 bp, paired-end reads.

Raw metagenomic reads were processed using the SMRTlink 5.0 software with the following parameters: “–min_length 200, –max_drop_fraction 0.8, –no_polish TRUE, –min_zscore −9999, –min_passes 1, –min_predicted_accuracy 0.8, –max_length 18,000.” Human DNA was removed using Bowtie2 (v.2.5.1) by mapping the reads against the corresponding reference genomes. Taxonomic profiling was performed using MetaPhlAn (v4.0.6), with the CHOCOPhlAn database (vOct22); relative abundances of taxa were provided based on approximately 5.1 million unique clade-specific marker genes from around 1 million microbial genomes. Functional profiling of metagenomes was done using HUMAnN (v3.8). Reads were mapped to sample-specific pangenomes, including all gene families of detected microorganisms, using Bowtie2. Unmapped reads were aligned against UniRef90 using DIAMOND (v2.1.8). Hits were counted per gene family and normalized for length and alignment quality. UniRef90 abundances from nucleotide and protein levels were mapped to level 4 Enzyme Commission (EC) nomenclature and integrated into MetaCyc pathways.

### Biofilm experiments

*S. mutans* UA159 and *S. sanguinis* ATCC10556 were used in this study. Both were cultured statically in liquid or agar-solidified brain heart infusion (BHI) broth (Becton, Dickinson and Company, Franklin Lakes, NJ, USA) under aerobic conditions at 37 °C, except for *S. mutans*, which required an anaerobic environment on agar. Cells were harvested at the early stationary phase, washed twice with phosphate-buffered saline, and used for biofilm experiments. *S. mutans* were stained with 2 µL/mL of CellTrace stain (CFSE[carboxyfluorescein-diacetate-succinimidyl ester] green), whereas *S. sanguinis* was stained with CellTrace stain (far red), as described previously [41]. Mono-species (10^8^ cells/mL) and dual-species (each 10^8^ cells/mL) biofilms were cultivated aerobically at 37 °C in BHI supplemented with 0.8% glucose (BHIG) or fructose (BHIF) for 20 h using a saliva-coated well of an eight-well chamber slide system (Thermo Fisher Scientific, Waltham, MA, USA). The saliva coating was prepared with whole saliva collected from eight healthy donors, treated with 2.5 mM dithiothreitol, clarified, diluted to 25%, and filter-sterilized, as previously described [42, 43]. BHI contains 0.2% glucose, yielding final concentrations of 1.0% glucose in BHIG and 0.2% glucose with 0.8% fructose in BHIF. After gentle washing, the biofilm microstructure was visualized using a confocal laser scanning microscope (Leica SP8; Leica Microsystem, Wetzlar, Germany) and analyzed with Imaris software (v10.1.1; Oxford Instruments, Abingdon, UK).

### Statistical analysis

Principal component analysis (PCA) was conducted using SIMCA (v16; Sartorius AG, Goettingen, Germany) to compare metabolomic profiles of plasma, glandular saliva, and whole saliva. PCA score plots were Pareto-scaled. We performed omnibus testing with permutational multivariate analysis of variance (PERMANOVA) to quantify the percentage of variance in each metabolomic profile explained by cardiometabolic parameters. This was performed using the Bray–Curtis dissimilarity metric and adonis function in the R package vegan 2.6–4. To evaluate the association between glycemic parameters and metabolomic saccharide abundances, Spearman’s rank correlation was performed using GraphPad Prism software (v.8). All *p*-values from the PERMANOVA and Spearman’s rank correlation were corrected for multiple comparisons using the Benjamini–Hochberg procedure via GraphPad Prism software (v.8). For saccharide migration analysis, participants were categorized into septiles based on glucose, fructose, and their combined levels in metabolomic profiles, ranking plasma, glandular saliva, and whole saliva levels of glucose, fructose, and their total from lowest to highest (scores 1 to 7). The saccharide migration score (3 to 21) was calculated by summing the scores for glucose (GlcMig), fructose (FruMig), and their total (GlcFruMig) across all biofluids, with higher scores indicating greater saccharide migration. The association between oral clinical parameters and the saccharide migration score was assessed using the lm() function in R, with the saccharide migration score as the dependent variable and age and gender as covariates to evaluate the effect size of each clinical parameter.

For metagenomic per-feature testing, taxonomic and functional features underwent quality control filtering. Features were excluded if they did not meet a minimum prevalence threshold (10% of samples) or relative abundance threshold (0.1% for microbial species, 0.001% for pathways, and 0.00001% for enzymes). Additionally, highly correlated functional features were filtered by retaining the most abundant feature in each cluster (cross-sample Spearman correlation > 0.9). After filtering, 120 microbial species, 246 pathways, and 1575 enzymes were included in the analyses. This prefiltering was applied to mitigate the effect of zero-inflation and the burden of multiple-testing, as well as to improve power and estimate stability.

We used a multivariate linear model in MaAsLin 2 [44] to identify features associated with glycemic parameters and saccharide migration scores. Each parameter was analyzed separately, adjusting for age, severity of periodontitis, extent of caries, and salivary flow as covariates. Participants were categorized into tertiles based on age, PISA, and the number of carious teeth with ICDAS scores of ≥ 3. Salivary flow was categorized into low and high flow groups using a cutoff of 0.67 mL/min. This threshold was selected to reflect the typical central tendency of unstimulated whole saliva (~ 0.6 mL/min) [45] and ensure a balanced categorization. To identify microbiome and metabolomic features associated with improved glycemic control following intensive treatment in patients with T2D, MaAsLin 2 models were fitted to compare pre- and post-treatment features, with patient ID as a random effect. All high-dimensional tests were corrected for multiple comparisons by controlling the false discovery rate using the Benjamini–Hochberg method with a target *q*-value of 0.25 (MaAsLin 2 default). This approach is widely used in discovery-phase microbiome studies to minimize false negatives in high-dimensional settings.

## Results

### Study description

We conducted a multi-omic prospective observational study comparing individuals with T2D and normoglycemic participants (Supplementary Data 1). This study expands upon previous research that focused on a subset of the data [28] by incorporating a newly developed metabolomic analysis of glandular saliva (Supplementary Fig. 1), along with metagenomic analysis of supragingival plaque samples. We analyzed 366 clinical samples, consisting of 91 whole saliva and supragingival plaque samples, and 92 plasma and glandular saliva samples from 31 patients and 30 systemically healthy controls. Samples from patients with T2D were collected at baseline and after 2 weeks of treatment. However, one patient was excluded from the post-treatment analysis due to incomplete collection of whole saliva and supragingival plaque, with minimal impact on statistical power. Ultimately, metagenomic data were generated for 91 plaque samples, whereas metabolomic data were obtained for 91 whole-saliva samples and 92 plasma and glandular saliva.

### Plasma-to-saliva migration of saccharides

**Fig. 1:**
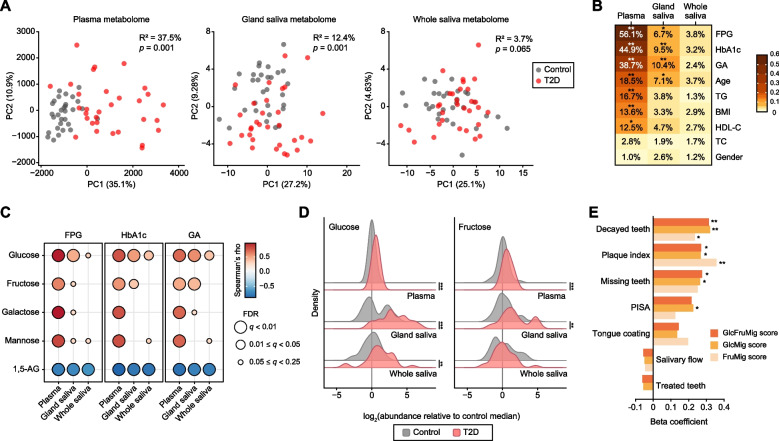
Saccharide migration from plasma to saliva and its impact on oral health. **A** Principal component analysis score plots illustrating metabolomic profiles of plasma, glandular saliva, and whole saliva, along with PERMANOVA results for Bray–Curtis dissimilarities based on T2D diagnosis. **B** Proportion of variation in metabolomic profiles explained by covariates and circulating biomarkers, quantified by PERMANOVA of Bray–Curtis dissimilarity. Stars show the *q*-values (false discovery rate-adjusted *p*-value) calculated using the Benjamini–Hochberg method. **q < 0.01, *q < 0.05. **C** Associations between glycemic parameters and major monosaccharides shared across plasma, glandular saliva, and whole saliva. Bubble color and size indicate effect estimates and statistical significance, respectively, with multiple comparisons adjusted as above. **D** Glucose and fructose levels in plasma, glandular saliva, and whole saliva were compared between participants with T2D and those with normoglycemia using the Mann–Whitney *U* test. ***p < 0.001, **p < 0.01. **E** Associations of plasma-to-saliva migration of glucose and fructose with oral health. Beta coefficients were derived from linear regression models using composite scores that summarized plasma-to-saliva migration levels of glucose (GlcMig score), fructose (FruMig score), and their sum (GlcFruMig score), with age and gender as covariates. **p < 0.01, *p < 0.05. HbA1c, glycated hemoglobin: FPG, fasting plasma glucose; GA, glycated albumin; TG, triglyceride; HDL-C, high-density lipoprotein cholesterol; TC, total cholesterol; PISA, periodontal inflamed surface area

To investigate the hyperglycemia-induced migration of circulating metabolites into the oral cavity, we compared the metabolomic profiles of plasma, glandular saliva, and whole saliva between normoglycemic controls and patients with T2D at baseline. Our untargeted GC–MS/MS platform identified 78, 125, and 143 annotated hydrophilic metabolites in plasma, glandular saliva, and whole saliva respectively, with 53 shared among all three. PCA score plots showed glycemic status differences, with plasma reflecting the most distinct variations, followed by glandular saliva and whole saliva (Fig. 1A). Bray–Curtis dissimilarity-based PERMANOVA revealed that T2D diagnosis was associated with a higher percentage of variation in metabolomic profiles in the order of plasma (*R*^2^ = 37.5%), glandular saliva (*R*^2^ = 12.4%), and whole saliva (*R*^2^ = 3.7%), with whole saliva differences being insignificant (Fig. 1A). The same trend was observed for common glycemic parameters like fasting plasma glucose, glycated hemoglobin, and glycated albumin (Fig. 1B). These results indicate that the glandular saliva exhibits an intermediate metabolomic profile between these for plasma and whole saliva, making it a more sensitive indicator of clinical glycemic phenotypes than whole saliva.

Among the major monosaccharides included in the 53 shared hydrophilic metabolites, glucose and fructose showed decreasing positive correlations with glycemic parameters in the order of plasma, glandular saliva, and whole saliva. Conversely, 1.5-anhydroglucitol (1,5-AG) consistently showed a negative correlation (Fig. 1C). Case–control comparisons confirmed a similar trend in glucose and fructose levels (Fig. 1D), suggesting their migration from systemic circulation to the oral cavity and subsequent consumption by oral microbiota.

To further assess the impact of plasma-to-saliva saccharide migration on oral health, we developed a composite saccharide migration score for glucose (GlcMig score), fructose (FruMig score), and their sum (GlcFruMig score). Participants were ranked into septiles for glucose, fructose, and glucose + fructose levels measured in plasma, glandular saliva, and whole saliva. The three ranks were then summed to generate composite score, with higher values indicating greater saccharide migration. Biologically, the score approximates the amount of host-derived fermentable saccharides reaching the tooth surface by integrating systemic supply (plasma), glandular secretion (glandular saliva), and residual levels after intraoral microbial consumption (whole saliva). Including glandular saliva enhances the assessment of systemic-to-oral flux. After adjusting for age and gender, we found that saccharide migration was significantly higher in participants with dental caries and plaque accumulation (Fig. 1E).

### Supragingival microbiome changes linked to saccharide migration

**Fig. 2:**
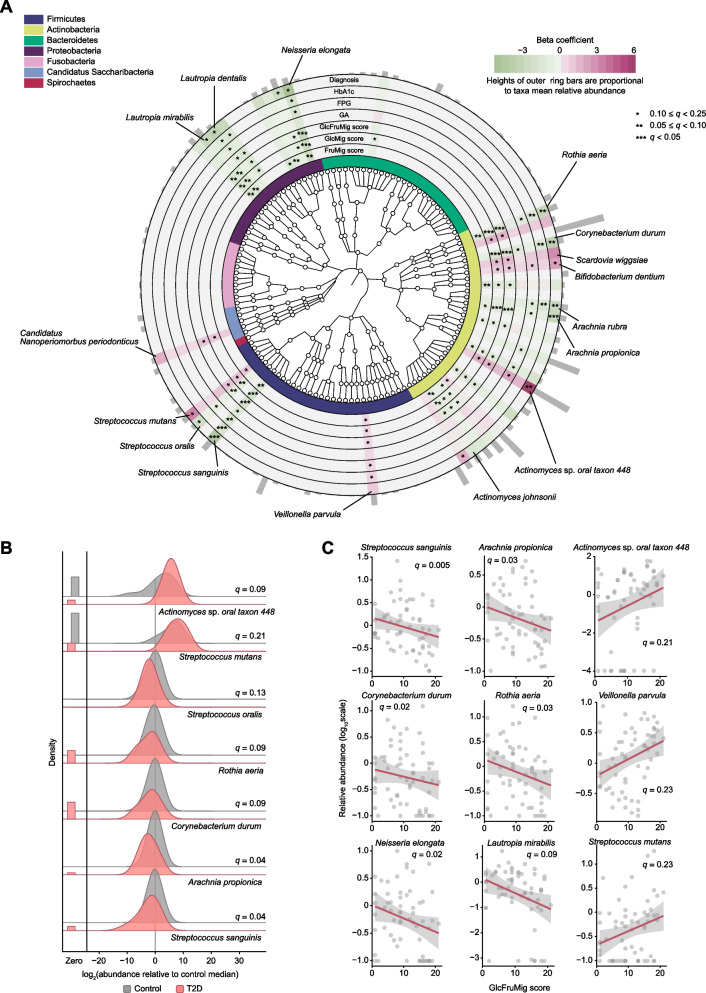
Supragingival microbial signatures linked to plasma-to-saliva saccharide migration. **A** Phylogenetically diverse oral microbial species significantly associated with T2D and saccharide migration. The green-to-red gradient represents the magnitude and direction of the associations quantified by linear mixed models that included each parameter as the independent variable and species abundance as the dependent variable. All models were adjusted for age, salivary flow, the extent of caries, and the severity of periodontitis. The colors of the innermost ring differentiate major phyla. The heights of the outermost bars are in proportion to the mean relative abundance of microbial species. The *q*-values (false discovery rate-adjusted *p*-value) were calculated using the Benjamini–Hochberg method with a target rate of 0.25. **B** Relative abundance of species significantly differing between participants with T2D and those with normoglycemia. **C** Significant associations between plasma-to-saliva migration levels of glucose and fructose (GlcFruMig score) with microbial species. The *q*-values in (**B**) and (**C**) were derived from multivariable-adjusted linear mixed models as above (T2D diagnosis and GlcFruMig scores, respectively), with multiple comparisons adjusted as above. Full results are provided in Supplementary Data 2–8. HbA1c, glycated hemoglobin: FPG, fasting plasma glucose; GA, glycated albumin

We next investigated changes in supragingival microbial composition associated with plasma-to-saliva saccharide migration. Bray–Curtis dissimilarity-based beta diversity of the supragingival microbiota differed between patients with T2D and controls (*R*^2^ = 6.2%; Supplementary Fig. 2), whereas Shannon and inverse Simpson index-based alpha diversity did not show significant variation (Supplementary Fig. 3). A total of 120 microbial species met the minimum prevalence (10% of samples) and relative abundance (0.1%) thresholds for inclusion in further analysis. Using multivariate linear models in MaAsLin 2, we identified microbial species associated with glycemic parameters and saccharide migration scores. All models were adjusted for potential confounders, including age, salivary flow, the extent of caries, and the severity of periodontitis (Supplementary Data 2–8). A total of 32 species-level features across five phyla were associated with at least one of the measures of hyperglycemia and saccharide migration (Fig. 2A). *Streptococcus mutans* and *Actinomyces* sp. *oral taxon 448* were enriched in diabetes, whereas *Streptococcus sanguinis*, *Arachnia propionica*, *Corynebactrium durum*, and *Rothia aeria* were depleted (Fig. 2A and B). Likewise, saccharide migration scores were positively associated with several caries-related species, including *S. mutans*, *Veillonella parvula*, *Actinomyces* sp. *oral taxon 448*, *Scardovia wiggsiae*, and *Bifidobacterium dentium*. However, these scores were negatively associated with *S. sanguinis*, *Streptococcus oralis*, *C. durum*, *R. aeria*, and several species of *Arachnia*, *Lautropia*, and *Neisseria* (Fig. 2A and C). The enrichment of *Nanoperiomorbus periodonticus*, a species within the Saccharibacteria (formerly TM7) phylum, was linked to saccharide migration and periodontitis severity (Fig. 2A and Supplementary Data 2–8). Furthermore, *Propionibacterium acidifaciens*, though not associated with saccharide migration, showed a strong correlation with the extent of caries (Supplementary Data 2–8). Among *Actinomyces* species, we noted variability in their associations: *Actinomyces gerencseriae* and *Actinomyces oris* were enriched, whereas *Actinomyces johnsonii*, *massiliensis*, and *naeslundii* were depleted.

**Fig. 3:**
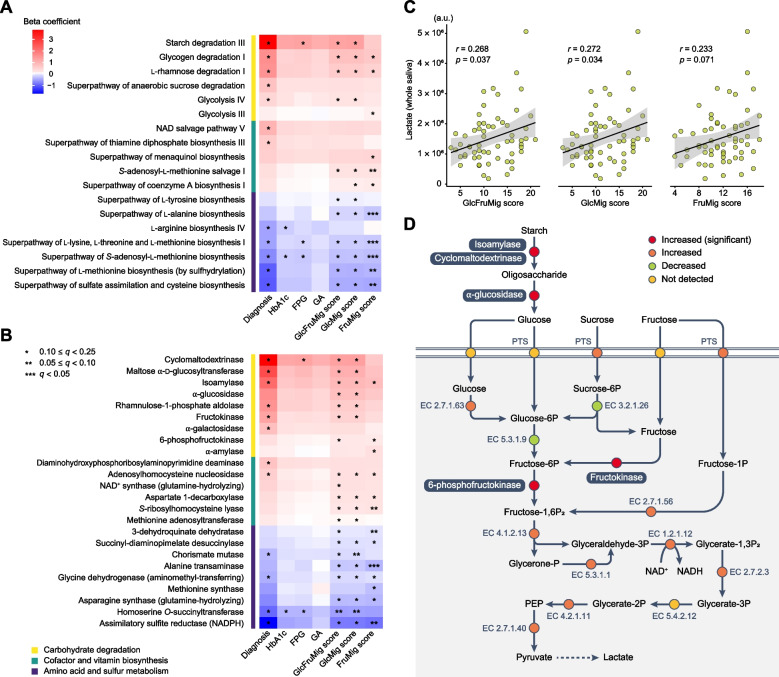
Community-level microbial functional potentials of supragingival biofilms associated with plasma-to-saliva saccharide migration. **A**, **B** Associations of T2D and saccharide migration scores with microbial functions (as MetaCyc pathways and EC enzymes). Beta coefficients were derived from multivariable-adjusted linear mixed models that included each parameter as the independent variable and the microbial pathway or enzyme abundance as the dependent variable. All models were adjusted for age, salivary flow, the extent of caries, and the severity of periodontitis. The *q*-values (false discovery rate-adjusted *p*-value) were calculated using the Benjamini–Hochberg method with a target rate of 0.25. A total of 18 pathways and 24 enzymes associated with at least one of seven parameters were grouped into carbohydrate degradation, cofactor and vitamin biosynthesis, and amino acid and sulfur metabolism. Full results are provided in Supplementary Data 9–22. **C** The correlations between saccharide migration scores and whole-salivary lactate levels. Spearman’s coefficients (*r*) and *p*-values are shown. **D** A schematic illustration of the enhanced community-level functional potentials of supragingival biofilms for carbohydrate degradation and glycolysis. Key enzymes significantly associated with saccharide migration are highlighted in white text on a dark background. HbA1c, glycated hemoglobin; FPG, fasting plasma glucose; GA, glycated albumin; PTS, phosphotransferase system; PEP, phosphoenolpyruvate

Next, we assessed changes in microbial functional potential associated with saccharide migration by analyzing MetaCyc pathways and EC enzyme abundances quantified from metagenomes, using MaAsLin 2. All models were adjusted for the same potential confounders as in the taxonomic analyses. A total of 18 pathways and 24 enzymes were associated with at least one of the measures of hyperglycemia and saccharide migration. These microbial changes generally reflected enrichments of community-level carbohydrate degradation and cofactor and vitamin biosynthesis, as well as depletion for amino acid and sulfur metabolism (Fig. 3A; Supplementary Data 9–15 and Fig. 3B; Supplementary Data 16–22). Specifically, the GlcFruMig score was positively associated with microbial pathways involved in carbohydrate degradation, including starch degradation Ⅲ (PWY-6731), glycogen degradation Ⅰ (GLYCOCAT-PWY), and Glycolysis Ⅳ (PWY-1042). Correspondingly, enzymes within these pathways, such as cyclomaltodextrinase (EC 3.2.1.54), isoamylase (EC 3.2.1.68), α-glucosidase (EC 3.2.1.20), fructokinase (EC 2.7.1.4), and 6-phosphofructokinase (EC 2.7.1.11), were also enriched. Similarly, NAD salvage pathway Ⅴ (PWY3O-4107) and its enzyme NAD synthase (EC 6.3.5.1) were more abundant in participants with greater GlcFruMig score, suggesting a higher demand for NAD, a hallmark of upregulated glycolysis. Furthermore, a significant correlation was observed between the GlcFruMig score and whole-salivary lactate levels, which serve as a community-level indicator of plaque acidogenesis (Fig. 3C). These results suggest that plasma-to-saliva saccharide migration enhances the functional potentials of supragingival biofilms for carbohydrate degradation and glycolysis, promoting disease-related acidogenesis (Fig. 3D).

### Effect of intensive glycemic control on saccharide migration and supragingival microbiome

**Fig. 4:**
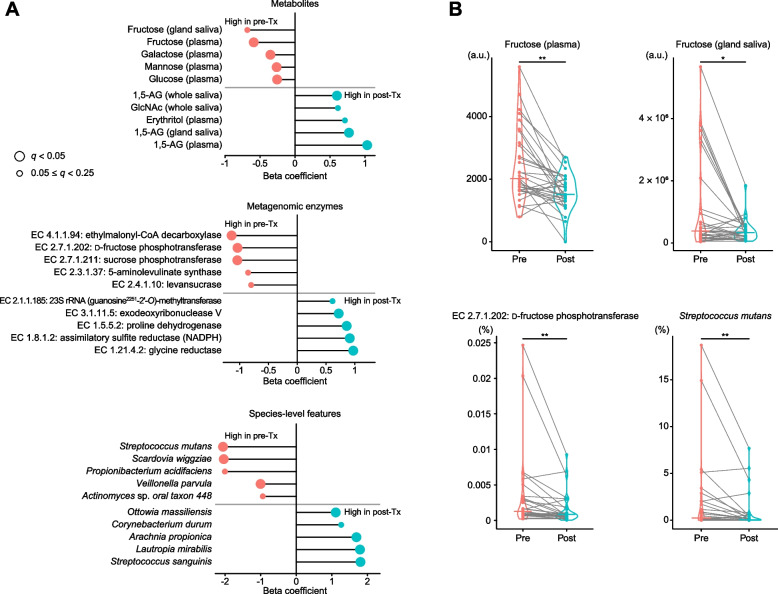
Effect of intensive glycemic control on saccharide migration and supragingival microbiome. **A** Metabolomic and microbiome changes following improved glycemic control in patients with T2D. Beta-coefficients of top features from each MaAsLin 2 model are presented, comparing pre- and post-treatment features with patient ID as a random effect. Bubble color and size indicate the direction of associations and *q*-values, respectively, (false discovery rate-adjusted *p*-value) calculated using the Benjamini–Hochberg method with a target rate of 0.25. Full results are provided in Supplementary Data 23–25. **B** Changes in plasma-to-saliva fructose migration, *Streptococcus mutans* abundance, and community-level fructose phosphotransferase carriage in the supragingival microbiome before and after glycemic control in T2D patients are illustrated. Dots represent patient values, with lines linking pre- and post-treatment data. Violin plot central lines indicate medians, and stars denote *q*-values from multivariable-adjusted linear mixed models with multiple comparisons adjusted as above. **q < 0.05, *0.05 ≤ *q* < 0.25. GlcNAc, *N*-acetylglucosamine; 1,5-AG, 1,5-anhydroglucitol; Tx, treatment

Finally, we examined the microbiome and metabolomic features altered by improved glycemic control through intensive treatment for patients with T2D. As previously described [39], glycemic control treatment significantly reduced the levels of HbA1c (from 9.1% to 8.4%), GA (from 23.7% to 19.4%), and FPG (from 8.1 to 6.1 mmol/L). Using MaAsLin 2 models comparing pre- and post-treatment features with patient ID as a random effect, we found that improved glycemic control significantly decreased plasma levels of several monosaccharides, with fructose showing the most prominent reduction in both plasma and glandular saliva (Fig. 4; Supplementary Data 23–25). This was accompanied by a significant reduction in supragingival community-level microbial carriage of fructose phosphotransferase (EC 2.7.1.202), essential for fructose uptake. Additionally, we observed decreased levels of cariogenic species like *S. mutans*, *S. wiggziae*, and *Propionibacterium acidifaciens*, and increased levels of *S. sanguinis*, *Lautropia mirabilis*, and *Arachnia propionica*, with the most prominent changes occurring in the streptococcal profiles (Fig. 4A and B). These results suggest that improved glycemic control reduces the influx of monosaccharides, particularly fructose, into the oral cavity, yielding microbial taxonomic and functional changes, including decreased carriage of the fructose uptake system and reversed abundances of *S. mutans* and *S. sanguinis* in the supragingival microbiome.

### Effect of exogenous fructose on *S. mutans*-*S. sanguinis* interactions in the mixed-species biofilm

**Fig. 5:**
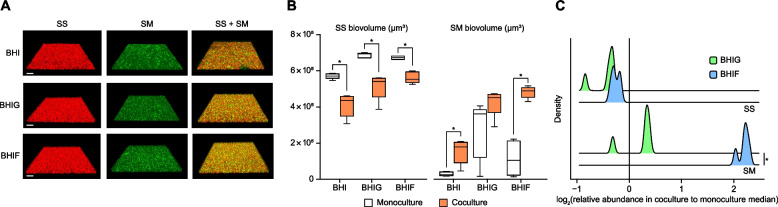
Effect of glucose and fructose supplementation on *S. mutans*-*S. sanguinis* interactions in mixed-species biofilms. **A** Representative confocal images of mono- and dual-species biofilms formed by *S. sanguinis* (red) and/or *S. mutans* (green). Biofilms were aerobically formed at 37 °C in brain heart infusion (BHI) supplemented with 0.8% glucose (BHIG) or fructose (BHIF) for 20 h using a saliva-coated well of an eight-well chamber slide system. Scale bars, 40 µm. **B** Biovolume of mono- and dual-species biofilms of *S. mutans* and *S. sanguinis*, based on computational image analysis of panel (**A**). Biovolumes of each strain were compared between mono- and dual-species biofilms using the Mann–Whitney *U* test. *p < 0.05. **C** Enhanced *S. mutans* growth in coculture with *S. sanguinis* in BHIF compared to that in BHIG. Effects of glucose or fructose supplementation on *S. mutans* growth in coculture were compared using the Mann–Whitney *U* test. *p < 0.05. SS, *S. sanguinis*; SM, *S. mutans*

To assess the dysbiosis-causing potential of fructose, mono- and dual-species biofilms of *S. mutans* and *S. sanguinis* were cultured aerobically for 20 h in BHI supplemented with glucose or fructose. We selected this pair as it is a well-characterized antagonistic model central to supragingival caries ecology [14, 46]. Analysis of biofilm microstructures demonstrated that both conditions increased the biovolume of *S. mutans* in coculture (Fig. 5A and B). Notably, the growth increase in coculture compared to monoculture was significantly higher with fructose than with glucose (Fig. 5C). Given that BHI already contains 0.2% glucose, the enhanced growth observed under fructose conditions suggest that the combination of glucose and fructose further promoted *S. mutans* overgrowth in coculture with *S. sanguinis*. These findings imply that glycemic control mitigates supragingival dysbiosis by reducing fructose influx into the oral cavity, which otherwise disrupts the balance between *S. mutans* and *S. sanguinis*, promoting the development of dental caries.

### Age-balanced sensitivity analysis

Although our primary analyses were adjusted for age, we conducted an age-balanced subanalysis to further address the pronounced age difference between groups (controls 42.10 ± 11.24 years; T2D 63.87 ± 10.50 years). We established an age-matched subset (12 controls, 12 T2D; controls 53.42 ± 9.08 years; T2D 54.67 ± 12.03 years; Mann–Whitney *U* test, *p* = 0.7226) and investigated whether the saccharide-migration score (GlcFruMig) was associated with the *S. mutans*-to-*S. sanguinis* abundance ratio in models adjusted for age and gender. Notably, the association remained positive and of similar magnitude in the age-balanced subset (beta coefficient 0.5625, 95% CI 0.1856–0.9394, *p* = 0.00548) as in the full cohort (beta coefficient 0.5087, 95% CI 0.2641–0.7534, *p* = 0.000107), indicating that baseline age differences do not explain this relationship (Supplementary Fig. 4).

## Discussion

Our study demonstrates that hyperglycemia-induced saccharide migration from plasma to the oral cavity was associated with a supragingival microbiome enriched in cariogenic organisms carrying pathways related to glycolysis and carbohydrate degradation. Moreover, improved glycemic control significantly reduced this saccharide influx, particularly fructose, leading to a microbiome shift toward less acidogenic and aciduric organisms with diminished fructose uptake capacity. Although the epidemiological connection between T2D and dental caries is well-documented, the underlying biological mechanisms remain unclear, with the prevailing explanations centering on decreased salivary flow and increased dietary sugar intake [47]. Elevated saccharide levels in saliva, mirroring those in blood, may drive the outgrowth of cariogenic bacteria [23, 24]. However, empirical research testing this hypothesis has been scarce. Our study addresses this gap by providing the first comprehensive mechanistic evidence—using metabolomic and metagenomic approaches—to illustrate the critical role of systemic metabolic control influences in oral health.

Recently, salivary metabolites have gained attention as potential non-invasive biomarkers for systemic diseases like diabetes and atherosclerosis [26, 27, 29, 48, 49]. However, most studies have focused on whole saliva, which contains metabolites produced or modified by the oral microbiome. This focus restricts the ability to study the correlations between these metabolites and blood metabolites, as well as their interactions with the oral microbiome. A key strength of our study lies in comparing plasma, whole saliva, and glandular saliva metabolomic profiles. This approach enabled us to trace metabolite migration from circulation into the oral cavity and assess the impact of oral microbiota on metabolite composition. The study revealed that glandular saliva metabolomic profiles had intermediate dissimilarity between plasma and whole saliva, initially resembling plasma but changing upon entering the oral cavity. This aligns with previous findings that metabolomic profiles of whole saliva were less predictive of glycemic variation than plasma [28, 29]. Additionally, we identified glucose and fructose as circulating metabolites that migrate into the oral cavity and are consumed by oral microbiota during hyperglycemia. While glucose migration has been previously documented [23–25], our study provides the first evidence of fructose migration—likely facilitated by our use of glandular saliva samples. This process may be driven by hyperglycemia-induced microvascular damage and basement membrane changes, leading to increased permeability [50–52]. Although galactose and mannose are known to increase in the plasma of patients with T2D alongside glucose and fructose [53, 54], they showed weaker correlations with glycemic parameters in both whole and glandular saliva in our study. This suggests that salivary glands selectively diffuse certain saccharides or that additional microbial metabolism occurs in the oral cavity. Parotid glands—not examined in our study—may play a role in this process, as recent research indicates distinct metabolite profiles in parotid saliva [55] and spatial metabolomic variation across oral regions [56]. Beyond salivary secretion, gingival crevicular fluid (GCF), a serum-derived exudate that increases with periodontal inflammation, contains various metabolites [57] and may deliver them to the oral cavity. Elevated glucose levels in GCF during hyperglycemia [58] suggest that it could contribute to oral saccharide concentrations, although the extent of this contribution remains unclear. Research should further explore systemic-to-oral metabolite migration and the specific pathways involved.

Our study revealed significant associations between high plasma-to-saliva saccharide migration and dental caries, along with cariogenic taxonomic and functional shifts in the supragingival microbiome. For instance, *Streptococcus mutans*, a principal cariogenic pathogen, as well as newly recognized species like *Scardovia wiggsiae* and *Bifidobacterium dentium* [11, 12, 59, 60], were enriched in participants exhibiting increased saccharide migration. Similarly, *Veillonella parvula*, implicated in caries through consumption of lactate produced by acidogenic bacteria [61], was elevated. Additionally, *Propionibacterium acidifaciens*, which did not correlate with saccharide migration but strongly correlated with the extent of caries in our study, has been linked to deep dentin caries [62], suggesting that its enrichment reflects the microenvironment of established lesions. Meanwhile, some recently recognized cariogenic species, such as *Selenomonas sputigena*, *Prevotella salivae*, and *Leptotrichia wadei* [13], as well as *Candida albicans* [6, 63], were excluded from our analysis due to their failure to meet the minimum prevalence and relative abundance thresholds. Conversely, *Streptococcus sanguinis* and *Streptococcus oralis*, which were depleted in participants with higher saccharide migration in our study, are strongly correlated with oral health [12, 14] and inhibit *S. mutans* through H_2_O_2_ production [46]. Similarly, *Corynebacterium durum*, another health-associated species [15], is in close contact with *S. sanguinis* in supragingival biofilms [64] and enhances its fitness via glycerol cross-feeding [65, 66]. Additionally, we identified significant depletion of nitrate-reducing bacteria, such as *Rothia aeria*, *Neisseria elongata*, and *Arachnia propionica* (formerly *Pseudopropionibacterium propionica* [67]), all linked to oral health [68] and explored as anti-caries probiotics [69].

Another notable species that was linked to saccharide migration and increased in T2D was *Actinomyces* sp. *oral taxon 448*, a prevalent yet understudied member of supragingival plaque [70]. The abundance of this species increases in the dental plaques of patients with T2D [71]. It has also been linked to severe early childhood [62], coronal [72], dentin [73], and root caries [74]. Given the strong adhesive capacity of *Actinomyces* [75–77] and its high prevalence on root surfaces irrespective of caries [78–80], increased periodontitis-related root exposure in patients with T2D may promote its preferential colonization in the oral cavity, leading to enrichment in supragingival plaque. Although our study focused on coronal caries and supragingival plaque, future inclusion of root-caries outcomes and metagenomic profiling of exposed root surfaces may help clarify its role in T2D-related caries pathogenesis. This species also co-occurs with *S. mutans* [12, 81] and shares similar acidogenic and aciduric traits [11]. The latter study noted heterogeneity in the cariogenic potential of *Actinomyces* species, consistent with our findings of enriched *Actinomyces gerencseriae* and *Actinomyces oris*, and depleted *Actinomyces johnsonii*, *Actinomyces massiliensis*, and *Actinomyces naeslundii*. This interspecies variability may explain the conflicting evidence regarding positive and negative associations between *Actinomyces* genus enrichment and T2D [82]. Notably, the enrichment of *Nanoperiomorbus periodonticus* was associated with saccharide migration and periodontitis severity. This species belongs to the Saccharibacteria phylum (formerly TM7), where episymbiosis with oral *Actinomyces* has been demonstrated, particularly in the related TM7x (*Nanosynbacter lyticus*) lineage [63, 83]. Although it remains unclear whether *N. periodonticus* behaves similarly, its co-enrichment with saccharide migration suggests a possible expansion of the *Actinomyces*–TM7 consortium in response to increased saccharide availability. Compared to typical 16S rRNA analysis, our shotgun-metagenomic analysis revealed a higher prevalence of *Actinomyces*, corroborating findings from prior shotgun metagenomic research on supragingival plaques [84, 85]. This discrepancy likely reflects the amplification bias in 16S rRNA analysis, which underestimates high G + C content *Actinomyces* [85]. Additionally, swabbing methods commonly used for plaque collection may miss *Actinomyces* strongly adhered to the enamel base, unlike our Gracey curette approach, potentially skewing plaque profiles. Addressing these factors in future studies is crucial for accurate supragingival microbiome analysis.

Participants with higher saccharide migration exhibited community-level microbial functional shifts, including alterations in carbohydrate and glucose metabolism, upregulated biosynthesis of cofactors and vitamins, and downregulated sulfur metabolism, all previously associated with caries in microbiome studies [86, 87]. Notably, our study highlighted broader saccharide utilization by oral biofilms via enhanced carbohydrate degradation and glycolysis, consistent with a prior report of increased saccharide uptake in individuals with dental caries [86]. Additionally, the enrichment of two-component signaling systems in the caries microbiome, as previously reported [86], aligns with the observed upregulation of the S-adenosyl-L-methionine salvage pathway, which facilitates AI-2 quorum sensing. Together with the positive association between the saccharide migration scores and lactate measured in whole saliva, these results demonstrate how diabetes-associated molecular changes in saliva shape the taxonomic and functional profiles of oral biofilms, offering mechanistic insights into the relationship between diabetes and dental caries.

By enrolling a unique cohort of patients with T2D undergoing a 2-week intensive glycemic control regimen in a controlled hospital setting, we could collect detailed information on how improved glycemic control influences oral microbiome and metabolite changes, corroborating the link between diabetes and dental caries mediated through plasma-to-saliva saccharide migration. Importantly, reduced circulating fructose migration into saliva coincided with decreased carriage of fructose uptake functions, along with reduced cariogenic species and increased non-cariogenic species in the supragingival microbiome, suggesting that glycemic control may reduce the risk of caries in patients with T2D. These microbiome changes are consistent with the effect of improved glycemic control, as no oral hygiene instructions were given; plaque accumulation remained unchanged throughout the 2-week treatment [34]. Circulating fructose is often elevated in T2D [88], likely due to impaired hepatic uptake and metabolism of fructose and activation of the polyol pathway. Serum levels decline rapidly with improved glycemic control [89], aligning with our findings. The cariogenicity of fructose, a preferred carbon source for oral bacteria [90], has been confirmed in both animal models and humans [91, 92]. Recent studies have linked fructose consumption, particularly high-fructose corn syrup, to increased caries risk [93]. Fructose also enhances glucosyltransferase expression in *S. mutans* in the presence of sucrose, promoting biofilm accumulation and prolonged acidification [94]. Additionally, fructose uptake via the phosphotransferase system (PTS) generates methylglyoxal, impairing less tolerant *S. sanguinis* and enabling *S. mutans* to dominate in two-species communities [95]. These findings support our results, demonstrating the significant role of fructose in shaping caries-associated communities mainly by modulating oral streptococcal profiles.

Our study has several strengths. First, this is the first study to quantify the association between diabetes and dental caries using saccharide migration scores enabled by glandular saliva metabolomics. Second, it integrates metabolomics and shotgun metagenomics to uncover oral microbial taxonomic and functional changes influenced by plasma saccharide migration. Third, it provides empirical evidence that glycemic control reduces caries risk in patients with T2D, with fructose identified as a key modulator of the oral microbiome, moving beyond cross-sectional observations. These findings were supported by full-mouth examinations and thorough sampling. However, the present study has some limitations. Although our statistical models accounted for major confounders, the lack of comprehensive data on dietary habits and oral hygiene practices among patients with T2D prior to admission limited our ability to control for these covariates. This may influence oral saccharide levels and supragingival microbiome composition independently of T2D. Additionally, a larger sample size is needed to improve statistical power, enhance generalizability, and account for interindividual variation in the oral microbiome, which may have obscured subtle differences. Although we identified species and enzyme genes associated with saccharide migration, our study may have been underpowered to detect taxa and genes with smaller effect sizes. Therefore, larger cohorts are required to uncover these subtler associations (Supplementary Fig. 5). As prefiltering removes rare microbial features, we may have missed low-abundance taxa with meaningful effects. To fully evaluate their roles, larger cohorts, deeper sequencing, or targeted assays will be needed. Although baseline differences, particularly age, could confound our findings, the similar beta estimates observed in the age-balanced subanalysis suggest that age does not fully explain the association between saccharide migration and supragingival dysbiosis (Supplementary Fig. 4). Finally, some participants with T2D had poorly controlled diabetes before glycemic control treatment, complicating direct extrapolation to routine clinical populations.

## Conclusions

Our study identified alterations in supragingival microbiome composition and functions associated with the migration of circulating glucose and fructose from saliva into the oral cavity, providing evidence of plasma-to-saliva saccharide migration’s role in the pathogenesis of dental caries in patients with T2D. Furthermore, our findings indicate that glycemic control modulates these migration effects and the associated microbiome, with fructose emerging as a key metabolite shaping caries-associated biofilms. These findings deepen our understanding of the biological link between diabetes and dental caries, highlighting the potential effectiveness of glycemic control in preventing dental caries in patients with T2D.

## Supplementary Information

Supplementary Material 1: Supplementary Data 1.

Supplementary Material 2: Supplementary Figures 1-5.

Supplementary Material 3: Supplementary Data 2-8.

Supplementary Material 4: Supplementary Data 9-15.

Supplementary Material 5: Supplementary Data 16-22.

Supplementary Material 6: Supplementary Data 23-25.

## Data Availability

The shotgun metagenomic sequencing data is available in the NCBI sequence read archive (SRA) repository with the accession number PRJNA1225502, https://www.ncbi.nlm.nih.gov/bioproject/PRJNA1225502. Metabolomic datasets are available in the Metabolomics Workbench repository with the study ID ST001905, http://www.metabolomicsworkbench.org/data/DRCCMetadata.php?Mode = Study&StudyID = ST001905; and ST001906, http://www.metabolomicsworkbench.org/data/DRCCMetadata.php?Mode = Study&StudyID = ST001906.
