# Supplementary material for: Diabetes alters the supragingival microbiome through plasma-to-saliva migration of glucose and fructose
Source: Microbiome. 2025 Dec 4;14:48. doi: 10.1186/s40168-025-02256-x (PMC12849494; doi:10.1186/s40168-025-02256-x)
Supplement: Supplementary file 3 — Supplementary Material 2: Supplementary Figures 1-5. [file 40168_2025_2256_MOESM2_ESM.docx]

**Supplementary Material 2**


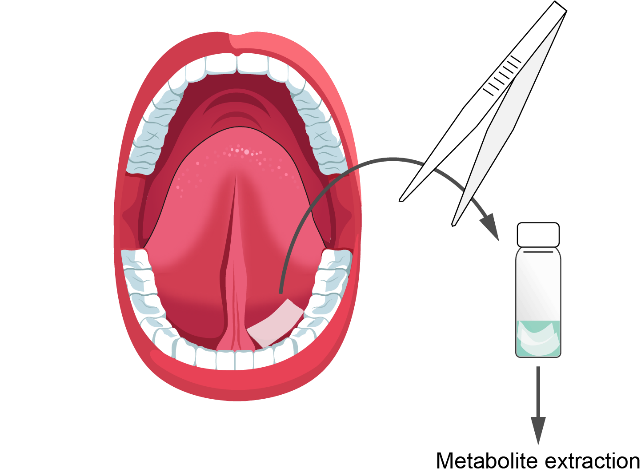


**Supplementary Figure 1.** Schematic illustration of glandular saliva collection. A filter paper was placed sublingually using tweezers while participants held their tongues against the palate to prevent contact with unintended areas. Once saturated, the filter paper was transferred into a 2-mL glass vial containing 530 µL of distilled water and an internal standard. This procedure was performed bilaterally, yielding two filter papers per vial.


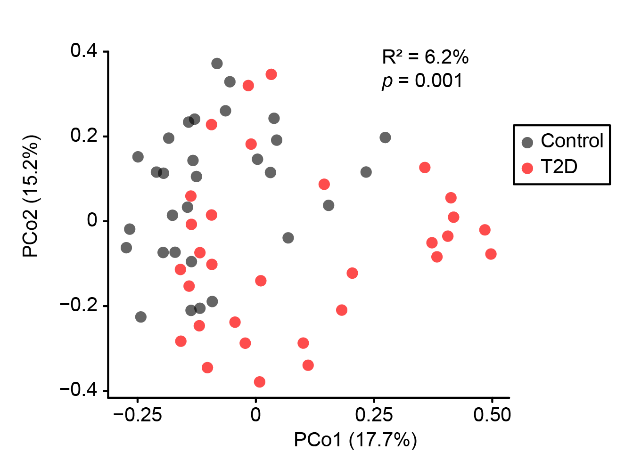


**Supplementary Figure 2.** Principal coordinate (PCo) analysis of the supragingival microbiome at the species level, based on Bray–Curtis dissimilarity and stratified by T2D status. PERMANOVA results revealed significant differences in the overall microbial community between participants with T2D and those who were normoglycemic.


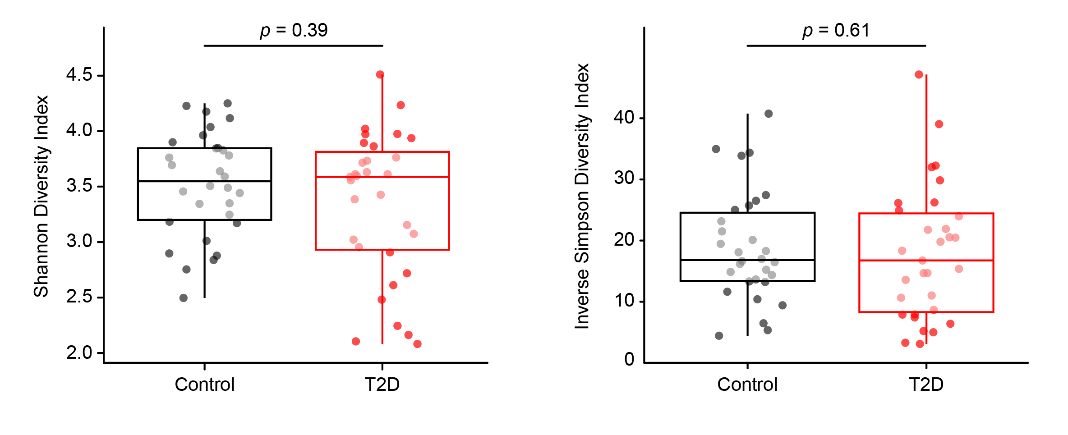


**Supplementary Figure 3.** Analysis of supragingival microbial diversity by T2D status at the species level. Differences in the distributions of the Shannon diversity index and the inverse Simpson index were assessed using Wilcoxon tests.


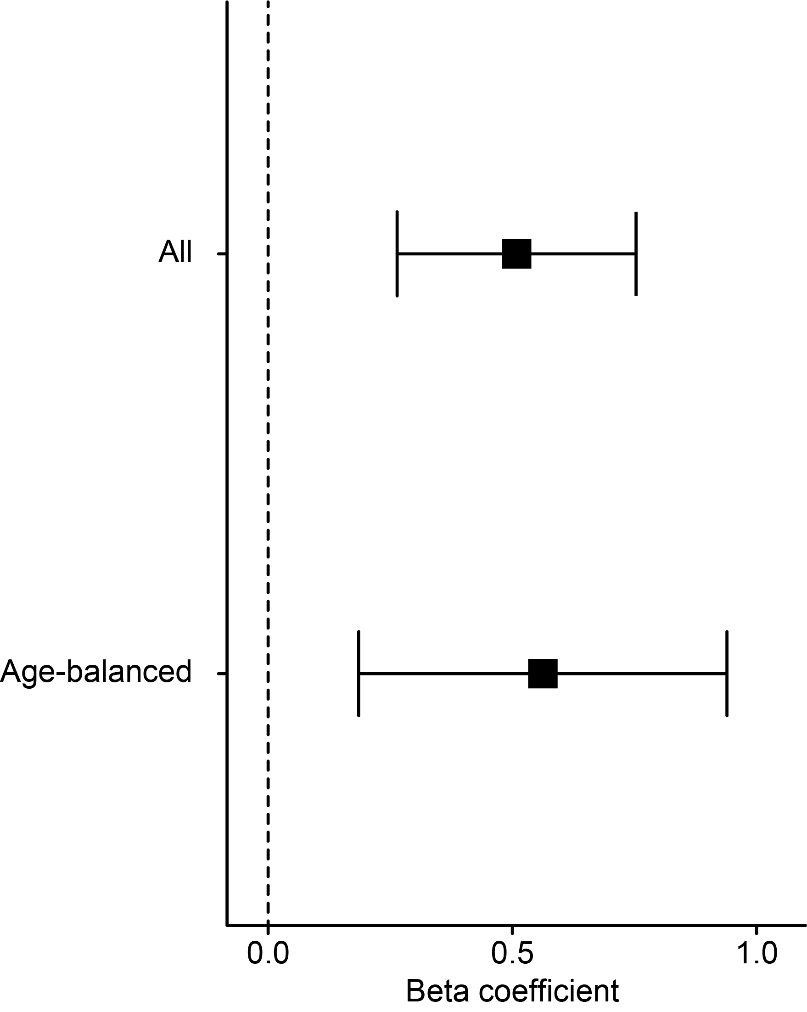


**Supplementary Figure 4.** Forest plots showing associations, in all participants and in an age-balanced subset, between plasma-to-saliva glucose and fructose migration (GlcFruMig score) and the log_10_-transformed *Streptococcus mutans*–to–*Streptococcus sanguinis* ratio. Boxes represent point estimates, and error bars denote 95% confidence intervals. Both models were adjusted for age and sex.

**
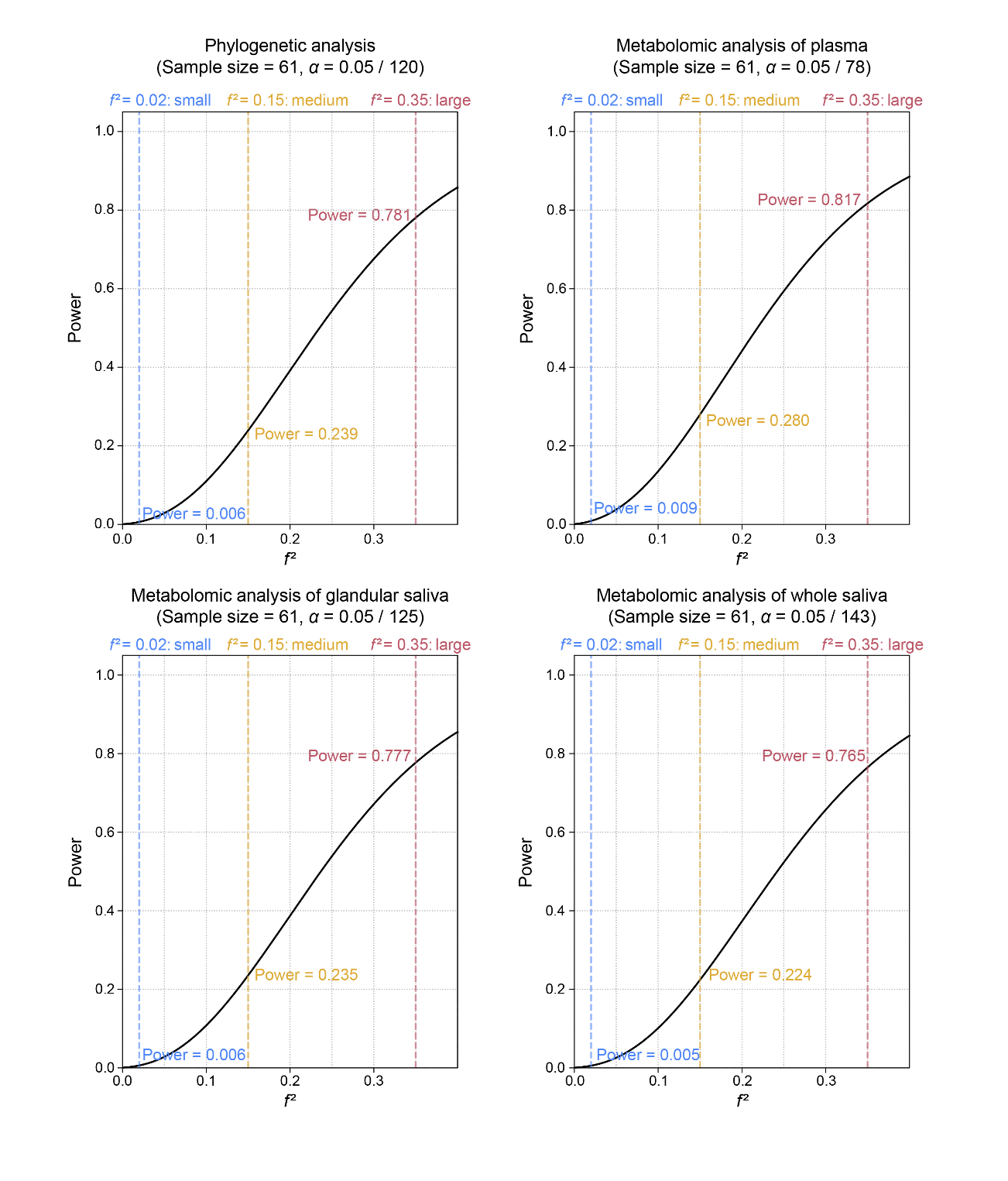
Supplementary Figure 5.** Statistical power analysis of the phylogenetic and metabolomic datasets. Power was calculated separately for each omics dataset. Small, medium, and large effect sizes are defined as *f*^2^ ≥ 0.02, *f*^2^ ≥ 0.15, *f*^2^ ≥ 0.35, respectively. *f*^2^, Cohen’s *f*^2^.
